# Potential Oral Health Care Agent from Coffee against Virulence Factor of Periodontitis

**DOI:** 10.3390/nu11092235

**Published:** 2019-09-16

**Authors:** Sing-Hua Tsou, Suh-Woan Hu, Jaw-Ji Yang, Min Yan, Yuh-Yih Lin

**Affiliations:** 1 Institute of Oral Sciences, College of Oral Medicine, Chung Shan Medical University, Taichung 40201, Taiwan; 2 School of Dentistry, College of Oral Medicine, Chung Shan Medical University, Taichung 40201, Taiwan; 3 Department of Stomatology, Chung Shan Medical University Hospital, Taichung 40201, Taiwan

**Keywords:** chlorogenic acid, *Porphyromonas gingivalis*, time-kill test, protease activity, disk-diffusion test

## Abstract

Background: Coffee is a major dietary source of polyphenols. Previous research found that coffee had a protective effect on periodontal disease. In this study, we aimed to investigate whether coffee extract and its primary phenolic acid, chlorogenic acid, affect the growth and protease activity of a periodontopathogen *Porphyromonas gingivalis* (*P. gingivalis*). Methods: Coffee extract and chlorogenic acid were prepared by a two-fold serial dilution. The turbid metric test and plate count method were used to examine the inhibitory effects of chlorogenic acid on *P. gingivalis*. The time-kill assay was used to measure changes in the viability of *P. gingivalis* after exposure to chlorogenic acid for 0–24 h. The protease activity of *P. gingivalis* was analyzed using the optical density of a chromogenic substrate. Results: As a result, the minimum inhibitory concentration (MIC) of chlorogenic acid was 4 mg/mL, and the minimum bactericidal concentration was 16 mg/mL. Chlorogenic acid at concentrations above MIC resulted in a longer-lasting inhibitory effect on *P. gingivalis* viability and significantly reduced associated protease activity. The coffee extract showed antibacterial activity as observed by the disk diffusion test, whereas these inhibitory effects were not affected by different roast degrees of coffee. ***Conclusions:*** Collectively, our novel findings indicate that chlorogenic acid not only has antimicrobial activity but also reduced the protease activity of *P. gingivalis*. In addition, coffee extract inhibits the proliferation of *P. gingivalis*, which may partly be attributed to the effect of chlorogenic acid.

## 1. Introduction

Periodontal disease is a common oral disease, affecting approximately 10–15% of adults worldwide, thus contributing to a large disease burden globally [1]. Periodontal disease represents an inflammatory disease and results in gingival recession, periodontal tissue destruction, and the loss of alveolar bone. One Gram-negative anaerobic bacterium, *Porphyromonas gingivalis* (*P. gingivalis*), is known to contribute to the risk of this disease [2]. Indeed, *P. gingivalis* is a major pathogen of periodontal disease, and it can colonize in subgingival sites, as well as invade periodontal tissue [2]. In addition, *P. gingivalis* has been shown to have a pivotal effect on the progression of periodontitis, despite low colonization levels at the lesion sites [3]. *P. gingivalis* colonization in subgingival sites may initiate the process of periodontal disease, subsequently activating other Gram-negative bacterial species [4]. Proteases of *P. gingivalis* are considered a virulence factor that may induce bacterial colonization and moderate the immune defense of the host [5]. Moreover, periodontal disease is one of the risk factors for systemic diseases, including adverse pregnancy outcomes, cardiovascular disease, and diabetes [6,7,8,9]. Thus, reducing the incidence and prevalence of periodontal disease may serve to decrease the overall probability of systemic diseases and the accompanying financial burden on worldwide health care systems [10].

Chlorogenic acid is a major phenolic acid in coffee and has been reported to be beneficial for human health due to its various biological activities, including antibacterial, antioxidant, anxiolytic, and anti-inflammatory activity, as well as its protective effect on cardiovascular disease and obesity [11,12,13,14]. Chlorogenic acid is relatively nontoxic in rats and dogs and there are no reports available with reference to man, other than some possible allergy reaction [15]. It has been investigated as to whether it is the causative agent in numerous herb-related instances of harm but does not appear to be causative [16]. A randomized control trial revealed that the number of oral bacterium *Streptococcus mutans* was reduced by green coffee extract, which has a high concentration of chlorogenic acid [17]. Other studies have also reported that green coffee extracts have efficacious antibacterial activity against periodontal pathogenic bacteria, with *P. gingivalis* being the most susceptible [18,19,20]. Despite these findings that the green coffee extract has antibacterial abilities, the effects of chlorogenic acid, as the major polyphenol in green coffee extract, on *P. gingivalis* and its protease activity are not fully understood [20,21]. Therefore, the aim of this study was to evaluate the antimicrobial efficacy of chlorogenic acid against *P. gingivalis*, particularly in regard to bacterial proteolytic activity.

## 2. Material and Methods

### 2.1. Chlorogenic acid

Chlorogenic acid (1,3,4,5-tetrahydroxy-cyclohexanecarboxylic acid 3-(3,4-dihydroxycinnamate) was purchased from Gold Biotechnology (St. Louis, MO, USA) and dimethyl sulfoxide (DMSO) was purchased from Merck Pvt. Ltd. (Selangor, Malaysia). We used 10% DMSO as a solvent to dissolve chlorogenic acid [22]. A stock solution of chlorogenic acid (128 mg/mL) was prepared in 10% DMSO. Chlorogenic acid solutions were stored in sterilized 24-well plates, which were later used for antibacterial tests. Different concentrations of the antibacterial test samples were prepared using a serial 1/2 dilution method, with the concentrations after dilution ranging from 2 to 64 mg/mL.

### 2.2. Bacterial Strain and Culture Conditions

The *P. gingivalis* strain ATCC 33277 was cultured anaerobically in Wilkins–Chalgren anaerobe broth (Oxoid, Hampshire, UK) and on Wilkins–Chalgren agar (Difco, Becton–Dickinson, and Co., France) at 37 °C. Aliquots of 100 μL bacterial medium were inoculated into 8 mL of growth medium in an anaerobic environment and kept at 37 °C overnight before experiments. An optical density of the bacterial solution of approximately 0.8–1 at a wavelength of 600 nm was chosen for this study, corresponding to 1 × 10^9^ colony forming units (CFUs) per mL.

### 2.3. Bacterial Activity Tests

Tests of the chlorogenic acid activity against *P. gingivalis* were accessed by turbidity measurement and the plate count method [23]. Visualizing the solution turbidity was used to determine the minimum inhibitory concentration (MIC) of chlorogenic acid. To dilute the stock solution of chlorogenic acid, 1 mL of 10% DMSO was added to the next six tubes separately. Then, 1 mL of the chlorogenic acid sample at 128 mg/mL was added to the initial tube containing 1 mL of 10% DMSO. This was considered to be a 1/2 dilution. The starting concentration of chlorogenic acid was 128 mg/mL with a 1/2 dilution, while the final concentration was 64 mg/mL. From a 1/2 diluted tube, 1 mL of chlorogenic acid was transferred to the second tube to make a 1/4 dilution. The serial dilution was repeated up to a 1/64 dilution for the chlorogenic acid solution. The concentrations of the chlorogenic acid solutions achieved by this serial dilution method were as follows: 64, 32, 16, 8, 4, and 2 mg/mL. All test tubes were prepared with 500 μL of bacterial inoculum mixed with either 500 μL of chlorogenic acid solution or 500 μL of control group solution. The final concentration of bacteria in each test tube was 5 × 10^4^ CFUs per 1 mL solution. The final concentrations of the chlorogenic acid in each test tube were 32, 16, 8, 4, 2, and 1 mg/mL, respectively. The control groups represented the negative control group as 10% DMSO, while 5.25% sodium hypochlorite represented the positive control group [24,25]. The MIC was determined after all tubes were incubated overnight at 37 °C in an anaerobic environment, and the tube of the lowest concentration of chlorogenic acid with a clear supernatant was taken as the MIC. A clear supernatant was defined as the black line in the background being distinctly observed. Conversely, if the black line in the background could not be clearly observed, it was defined as turbid solution. The clear supernatant was considered to represent no bacterial growth in the tube, while any cloudiness in the supernatant indicated that *P. gingivalis* could proliferate in that particular concentration of chlorogenic acid. The minimum bactericidal concentration (MBC) of chlorogenic acid was decided by counting the colony-forming units. Aliquots of 100 μL mixture solutions of chlorogenic acid and bacterial inoculum (1:1) were poured into and evenly smeared onto Wilkins–Chalgren agar plates before then being incubated at 37 °C for 24 h in an anaerobic environment. The lowest concentration of chlorogenic acid in which no apparent bacteria growth on the Wilkins–Chalgren agar occurred was designated as the MBC.

### 2.4. The Time-Kill Test

The killing kinetics of chlorogenic acid against *P. gingivalis* at concentrations of 32, 16, 8, and 4 mg/mL (2 × MBC, MBC, 2 × MIC, and MIC) were determined by the plate count method [26]. All test tubes were prepared with 500 μL of bacterial inoculum mixed with either 500 μL of chlorogenic acid solution or 500 μL of control group solution. The final concentration of bacteria in each test tube was 5 × 10^2^ CFUs per 1 mL solution. The control group represented the negative control group as 10% DMSO. All tubes were incubated at 37 °C in an anaerobic environment. Each sample was extracted after 3, 6, 9, 12, and 24 h incubation. At every time-point, an aliquot of 100 μL of each sample was evenly smeared onto the Wilkins–Chalgren agar plate and then incubated at 37 °C for 24 h in an anaerobic environment. The bacterial growth of each sample was determined by counting the colonies. This test was performed in triplicate.

### 2.5. Bacterial Protease Activity Test

Bacterial protease activity estimated as the optical density value of the hydrolysis of benzoyl-L-arginine ethyl ester hydrochloride (BANA) [27]. Briefly, one g BANA (Sigma-Aldrich, Co., St. Louis, MO, USA) powder was dissolved in 22.727 mL DMSO, and 100 mg of Fast Garnet GBC sulfate salt (Sigma-Aldrich, Co., St. Louis, MO, USA) powder dissolved in 100 mL Ethylene glycol monomethyl ether (Merck Pvt. Ltd., Selangor, Malaysia) was also prepared. BANA and the Fast Garnet GBC solution were stored at 4 °C. The effects of chlorogenic acid at concentrations of 32, 16, 8, and 4 mg/mL (2 × MBC, MBC, 2 × MIC, and MIC) on the protease activity of *P. gingivalis* were assessed using the BANA test. The test tubes were prepared with 500 μL of bacterial inoculum mixed with either 500 μL of the chlorogenic acid solution or 500 μL of the control group solution. The control group represented the negative control group as 10% DMSO. The final concentration of bacteria in each test tube was 5 × 10^4^ CFUs per 1 mL solution. All test tubes were incubated at 37 °C for 1 h in an anaerobic environment. The mixture solution (300 μL) was extracted from each tube and separately mixed with 300 μL of BANA solution, followed by the addition of 30 μL of Fast Garnet GBC solution in each tube. All tubes were incubated in a dark anaerobic environment at 37 °C for 30 min, and then the absorbance was measured using a micro-plate spectrophotometer (Epoch™, BioTek Instruments, Winooski, VT, USA) at 490 nm. The absorbance measurement method is how the optical density was measured. This test was performed in triplicate.

### 2.6. Coffee Preparation

A commercially available blend of both roasted and instant coffee extracts were used in the study (Moccona^TM^, New South Wales, Australia). The light-roast degree and dark-roast degree coffee extracts were chosen for the antibacterial test. Coffee extract samples for the test were prepared in 30 mL boiling sterilized double-distilled water and stored in sterilized 15 mL centrifuge tubes. The final concentrations of the coffee extract samples were 10, 5, 2, and 1 g in 30 mL sterilized double-distilled water, corresponding to 0.33, 0.17, 0.07, and 0.03 g/mL, respectively.

### 2.7. Disk Diffusion Test

The susceptibility of *P. gingivalis* to the coffee extracts at concentrations of 0.33, 0.17, 0.07, and 0.03 g/mL were assessed using the disk diffusion test [28]. A concentration of 10^5^ CFUs in 1 mL medium of bacteria was chosen for this test. Sterile cotton swabs were used to spread the bacterial inoculum onto Wilkins–Chalgren agar plates by the lawn of bacteria. Then six mm diameter filter paper discs were placed on Wilkins–Chalgren agar plates, and 20 μL of coffee extract samples were loaded onto the filter paper discs. Discs loaded with 20 μL of sterilized double-distilled water represented the negative control group. All plates were incubated in an anaerobic environment at 37 °C for 16 h until visible bacterial growth was observed. After 16 h incubation, inhibition zones formed around the filter papers discs. The mean of the diameters of these inhibition zones was calculated. This test was performed in triplicate.

### 2.8. Statistics

Statistical analysis was performed using JMP^®^ software (version 13.0, SAS Institute, Cary, NC, USA). All tests in this study were repeated at least twice. Data from the time-kill test were normally distributed, and two-way analysis of variance (ANOVA) with repeated measurements was used to evaluate the differences of the time effect between the experimental and control groups, as well as the differences between the treatment time-points of chlorogenic acid. Tukey’s honestly significant difference (Tukey’s HSD) was used to analyze which experiment groups may be different from the control group (10% DMSO as the negative control group in this test). For the BANA test, the mean percentage values between the experimental and control groups were tested for a significant difference by one-way ANOVA, while Tukey’s HSD was used to analyze which experiment groups of the mean percentage values may be different from the control group (the data of 10% DMSO of the negative control group was normalized as 100%). For the disk diffusion test, the inhibition zone of the light-roast degree and dark-roast degree coffee extracts were reported as the mean (with standard deviation). The significance level was set at *p* < 0.05.

## 3. Results

### 3.1. Antibacterial Activities of Chlorogenic Acid against P. gingivalis

#### 3.1.1. Bacterial Activity Test

**Figure 1:**
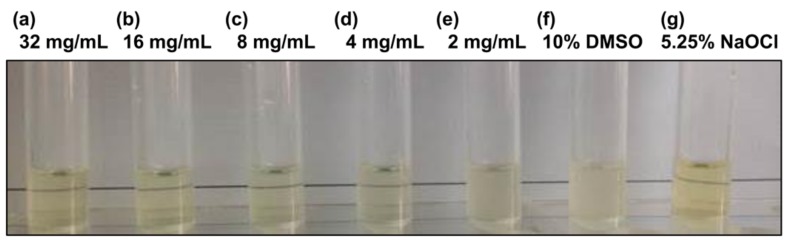
The minimum inhibitory concentration of chlorogenic acid against *Porphyromonas gingivalis*. The visualizing turbidity measurement method was used to determine the MIC. The test samples contained (**a**) 32 mg/mL, (**b**) 16 mg/mL, (**c**) 8 mg/mL, (**d**) 4 mg/L, and (**e**) 2 mg/mL chlorogenic acid dissolved in 10% dimethyl sulfoxide (DMSO). (**f**) 10% DMSO and (**g**) 5.25% sodium hypochlorite represented the negative and positive control groups, respectively.

**Figure 2:**
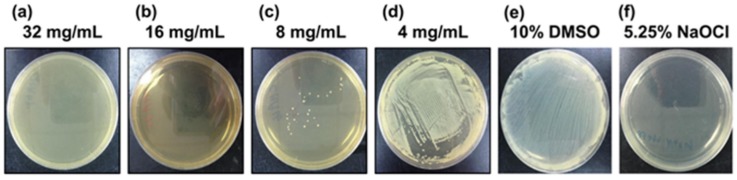
The minimum bactericidal concentration of chlorogenic acid against *Porphyromonas gingivalis*. The counting colony method was used to determine the MBC. Agar plates were treated with bacterial and (**a**) 32 mg/mL, (**b**) 16 mg/mL, (**c**) 8 mg/mL, and (**d**) 4 mg/mL chlorogenic acid dissolved in 10% dimethyl sulfoxide (DMSO), respectively. (**e**) 10% DMSO and (**f**) 5.25% sodium hypochlorite represented the positive and negative control groups, respectively.

We investigated the susceptibility of the *P. gingivalis* strain ATCC 33277 to chlorogenic acid. The MIC and MBC for chlorogenic acid are shown in Figure 1 and Figure 2. The MIC value of chlorogenic acid was 4 mg/mL (Figure 1), and the MBC value of chlorogenic acid was 16 mg/mL (Figure 2).

#### 3.1.2. Time Kill Test

**Figure 3:**
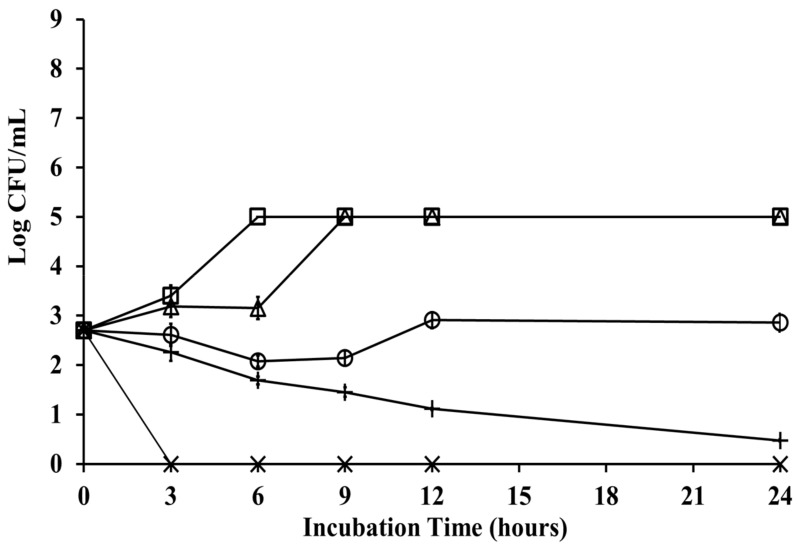
The time-kill effect of chlorogenic acid against *Porphyromonas gingivalis*. The cumulative change in the growth of the *P. gingivalis* strain after treatment with different concentrations of chlorogenic acid. The results show the antibacterial effect of chlorogenic acid at concentrations of (△) 2 mg/mL, (○) 4 mg/mL, (+) 8 mg/mL, (×) 16 mg/mL, and (□) 10% dimethyl sulfoxide of the negative control group, respectively. Chlorogenic acid at the MBC concentration showed a bactericidal effect after 3 h treatment.

Figure 3 shows the mode of growth and kill of *P. gingivalis* ATCC 33277 at different concentrations of chlorogenic acid. The time-kill assay indicated that *P. gingivalis* grew in 10% DMSO as the control group kept bacterial viability for at least 24 h incubation time. *P. gingivalis* treated with chlorogenic acid at increasing concentrations of 1/2 × MBC, and MBC (8 and 16 mg/mL) exhibited more than 90% of bacteria killed after 12 h of incubation. After a short lag phase, a slight decrease in bacterial viability was observed at 1/2 × the MBC concentration of chlorogenic acid after a 3 h treatment, achieving the lowest colony-formatting counts after 24 h of incubation. The bactericidal endpoint for *P. gingivalis* viability at the MBC concentration of chlorogenic acid was collected at 3 h time-points, with a reduction in CFUs by three log units (99.9%). Chlorogenic acid at the MIC concentration showed an inhibitory effect on *P. gingivalis* growth from 3 h onward, while bacteria escaped this effect after 12 h. *P. gingivalis* showed a lag phase of 3–6 h at 1/2 × the MIC, while a log phase was observed at 6–9 h. *P. gingivalis* treated with 1/2 × the MIC of chlorogenic acid showed a consistent performance of bacteria growth after 9 h incubation, compared with the control group.

#### 3.1.3. BANA Test

**Figure 4:**
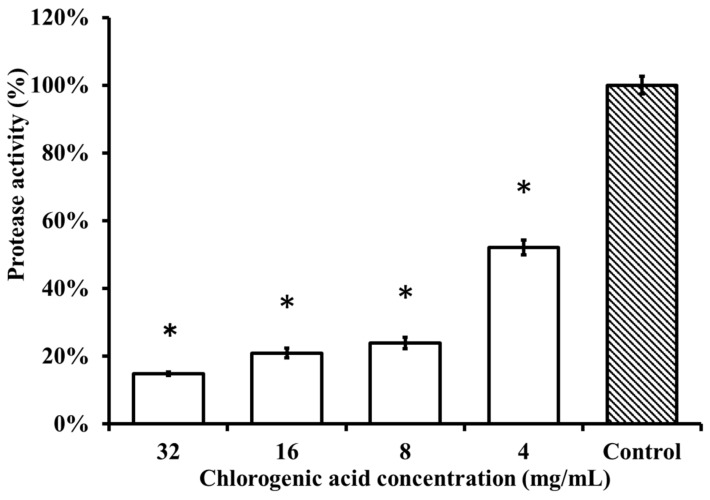
Chlorogenic acid inhibited the protease activity of *Porphyromonas gingivalis* (ATCC 33277) by the bacterial protease activity test. Percentage value changes in the protease activity of the *P. gingivalis* strain after treatment with chlorogenic acid at concentrations of 32, 16, 8, and 4 mg/mL, respectively, and treated with 10% dimethyl sulfoxide (DMSO) as the negative control group. The test indicated a significant difference between each concentration of the chlorogenic acid groups and 10% DMSO of the negative control group (* *p* < 0.001).

The protease activity of the *P. gingivalis* strain after treatment with chlorogenic acid at concentrations of 2 × MBC, MBC, 2 × MIC, and MIC (32, 16, 8, and 4 mg/mL) was significantly different from the control group, which was treated with 10% DMSO (*p* < 0.001) (Figure 4). Chlorogenic acid demonstrated a dose-dependent effect on the inhibition of *P. gingivalis* protease activity. The *P. gingivalis* strain treated with chlorogenic acid concentrations at MBC and 1/2 × MBC showed greater than a 70% reduction in protease activity compared with the control group.

### 3.2. Susceptibility of P. gingivalis to Coffee Extract

#### Disk Diffusion Test

Discs that were separately loaded with coffee extracts (0.03 g/mL, 0.07 g/mL, 0.17 g/mL, and 0.33 g/mL) had inhibition zones with diameters >6 mm (Table 1). The diameters of the inhibition zones ranged between 8.4 and 12.8 mm for the light-roast degree coffee extracts at concentrations between 0.07 and 0.33 g/mL, while the dark-roast degree coffee extracts at concentrations between 0.07 and 0.33 g/mL had inhibition zones with diameters ranging between 7.9 and 12.5 mm. Maximum activity was observed at the concentration of 0.33 g/mL of the light-roast degree coffee extract with a zone size of 12.8 mm. Coffee extracts were highly active against *P. gingivalis* at increasing concentrations and were shown to have dose-dependent effects against *P. gingivalis* in both the light-roast degree and dark-roast degree groups.

## 4. Discussion

*P. gingivalis* is a key pathogen in periodontal disease and has promotive effects that stimulate the host immune system to overproduce inflammatory cytokines [4,29]. In the present study, we investigated the effects of coffee extract and chlorogenic acid on the growth and protease activity of *P. gingivalis*.

Chlorogenic acid is a polyphenol that is found in fruits and vegetables; concentrations of chlorogenic acid are substantially greater in coffee [30,31,32]. Studies by Lou et al. and Saavedra et al. [33,34], have reported that chlorogenic acid has inhibitory effects on bacterial growth, including *Escherichia coli, Pseudomonas aeruginosa, Streptococcus pneumoniae, and Staphylococcus aureus.* However, the antibacterial effects of chlorogenic acid against *P. gingivalis* were not clearly defined in these studies. Our results demonstrated that *P. gingivalis* is indeed susceptible to chlorogenic acid. Karunanidhi et al. noted that an antibiotic-resistant bacterium, *Stenotrophomonas maltophilia*, was not only susceptible to chlorogenic acid but also showed a time-kill effect with chlorogenic acid treatment [26]. In this study, chlorogenic acid at the MBC concentration showed a prolonged killing effect on *P. gingivalis*. Chlorogenic acid at the MIC concentration inhibited bacterial growth until 9 h incubation, and bacteria regrew back to their initial number at 12 and 24-time points. The time-kill test indicated that the *P. gingivalis* response to chlorogenic acid was concentration-dependent. Although one study by Daglia et al. revealed that chlorogenic acid had weak inhibition activity against oral pathogenic bacteria, such as *Staphylococcus aureus* and *Streptococcus mutans* [35], our study clearly demonstrated that chlorogenic acid concentrations at the MIC value, or higher, inhibit *P. gingivalis* viability more than 50% from a 6 h incubation.

In addition to the viability of *P. gingivalis* at the lesion site, causing disease, associated protease activity may induce the process of periodontal disease development [3,36]. Proteases have been reported to break down host immune proteins and to interfere with other bacteria adhering to host tissue. In addition, cysteine proteases, such as Arg-gingipain, play an important role in *P. gingivalis* adhesion to the margin tissue of periodontal pockets [37,38]. Inaba et al. reported that the polyphenols of apple and hop bract inhibited the protease activity of *P. gingivalis* and reduced protease-induced down-regulation of enamel matrix derivative-stimulated tissue regeneration [39]. However, the effects of chlorogenic acid on the protease activity *of P. gingivalis* are not well understood. Therefore, we evaluated whether chlorogenic acid had effects on the activity of *P. gingivalis* proteases. According to our results of the time-kill test, chlorogenic acid at the MBC concentration showed no bacterial growth at the 3 h incubation time-point. Our results of the time-kill test showed that chlorogenic acid at the concentrations of 8 mg/mL or less had no effect on bacterial growth at 3 h time-point when compared to the negative control group (Figure 3). Our pilot experiments also indicated that the growth of *P. gingivalis* was not affected when bacteria were incubated with chlorogenic acid at a concentration of up to 32 mg/mL for 1 h. Thus, we investigated changes in the protease activity of *P. gingivalis* within an hour treatment of chlorogenic acid. Our study revealed that 4 mg/mL chlorogenic acid could reduce protease activity by more than 40%. Löhr et al. reported that a flower extract containing 1.6% chlorogenic acid could not downregulate the gene expression of Lys-gingipains [40], but they did not detect its protease activity. On the other hand, chlorogenic acid in our study at increasing concentrations was more effective in reducing the protease activity of *P. gingivalis.*

Chlorogenic acid can be found in certain plant species, including coffee, tea, cocoa, citrus fruits, berry fruits, apple, and pear with coffee containing the highest concentration [30]. The concentrations of chlorogenic acid range from 0.2 to 10 mg/mL that we usually intake from coffee. This large variation in chlorogenic acid content in the coffee beverages could be due to the differences in coffee species, planting, and harvesting conditions, bean preparation and roasting, and grinding and barista process. Whereas for tea, chlorogenic acid content is much lower than that in coffee, ranging from 0.0025 to 0.045 mg/mL [30]. The MBC concentration (16 mg/mL) of chlorogenic acid against *P. gingivalis* in our study is higher than the content in the coffee. Also, the MIC concentration (4 mg/mL) of chlorogenic acid and falls in the range of coffee we may consume in daily life. Coffee is still the best source of chlorogenic acid among beverages. A major source of chlorogenic acid can be found in green coffee extract, which is highly bioavailable to humans [41]. A study testing whether treatment outcomes for periodontal regeneration are influenced by loading green coffee extract onto Guided Tissue Regeneration (GTR) membranes indicated that green coffee extract showed no zone of inhibition against *P. gingivalis* [42]. The authors primarily indicated that GTR membranes loaded with green coffee extract had no preventive effect against *P. gingivalis* colonization. However, Mehta et al. found that in four plant and fruit extracts, coffee extract was the only one effective against *P. gingivalis* using the disk diffusion test [43]. Furthermore, Sage et al. found that chlorogenic acid is a major polyphenol among *A. borbonica* and could reduce the inflammatory response induced by *P. gingivalis* lipopolysaccharide [44].

Coffee extract is abundant in chlorogenic acid. However, some studies have revealed that the roast degree may influence the content of chlorogenic acid in the coffee extract [45,46]. Thus, we investigated whether the roast degree of the coffee extract affects the activity of *P. gingivalis*. According to the susceptibility test, we found that light-roast degree coffee extracts at increasing concentrations were the most effective in inhibiting the growth of *P. gingivalis*. Our results also indicated that the light-roast degree of coffee extract at the maximum concentration had the maximum activity on the susceptibility test of *P. gingivalis*. This is in accordance with a study by Yi et al., who further reported that medium-roast degree coffee extracts possessed antibacterial activity against *P. gingivalis* [28]. However, our study indicated that light-roast degree coffee extracts and dark-roast degree coffee extracts were nearly identical in the inhibition zone diameters of the *P. gingivalis* susceptibility test. Coffee extract inhibited bacterial growth, which may partly be attributed to the effect of chlorogenic acid. In this context, it is important to note that coffee extracts have recently been reported to be beneficial for oral health based on the high concentration of phenolic acids [47].

*P. gingivalis* strain ATCC 33277 has been reported to have a resistance effect on antimicrobial peptides [48]. Another study further suggested that the inhibition of protease activity may reduce bacterial pathogenicity, and this enzyme inhibitor may be a new therapeutic way for periodontal diseases [49]. A study showed that chlorogenic acid was effective in decreasing blood pressure in patients with mild hypertension, and that chlorogenic acid did not cause any apparent side effects in the patients [50]. However, this study was only focused on patients with mild hypertension, the side effects of chlorogenic acid on other groups of people have not yet been investigated comprehensively. Further research is required to elucidate the side effects of chlorogenic acid on other groups of people. Coffee is the major source of chlorogenic acid, which has been shown to have a protective effect on periodontal disease progression [20,51]. Coffee contains many nutrients, and the antibacterial activity of this solution may not directly be due to chlorogenic acid. However, chlorogenic acid could be considered as a potential oral health-promoting agent due to its antibacterial activity and the reduction of bacterial protease. *P. gingivalis* is a keystone bacterium in periodontitis-associated diseases, such as atherosclerosis, premature birth, and cancers [7,9,52]. Increasing evidences suggests that this oral bacterium has a role in oral cancer development [53,54,55]. The evidence linking oral microbiota with different types of cancer start to emerge. Cancer cells that had been chronically infected with *P. gingivalis* exhibited increased aggressiveness compared to noninfected cells [56] with higher metastatic potential and showed resistance to anticancer medicine, Taxol. Natural products derived from plants, such as chlorogenic acid, with a confirmed antibacterial properties against *P. gingivalis*, may play a role in the therapy or prevention of related cancers.

Bacteria in the oral cavity usually colonize on the surface of non-shedding hard tissues in the form of a biofilm and acquire characteristics different from planktonic cells [57]. As a result, the bacteria in biofilms are much more resistant to antimicrobial agents than cells in suspension [57]. It was noted that the effects of chlorogenic acid on *P. gingivalis* were examined in this study on planktonic bacterial cells, which was quite different from the clinical conditions. The effects of chlorogenic acid on bacterial adhesion and biofilm formation have not yet been elucidated. Further research is required to clarify the antibacterial effect of chlorogenic acid on biofilm. The current research focused on a single microbe, which is another limitation of this study. Since periodontitis is a polymicrobial infection in nature, other putative periodontopathogens may also play a pivotal role in the development of this disease. On that account, further research of chlorogenic acid effects on other oral bacteria is also a necessity. However, the results of the current study have already shed new light on the role of chlorogenic acid and possibly coffee in the process of *P. gingivalis* infection control during periodontitis development.

## 5. Conclusions

In summary, our study revealed a new method to apply the antibacterial characteristic of chlorogenic acid against *P. gingivalis* as an antimicrobial oriented clinical treatment. Collectively, our novel findings highlight the antibacterial efficacy of chlorogenic acid at 16 mg/mL against the periodontal disease pathogen *P. gingivalis* after 3 h treatment. Natural antimicrobial agents, such as chlorogenic acid, which is abundant in coffee and easily obtained by the general population, have antibacterial effects on *P. gingivalis*. Thus, chlorogenic acid or coffee may be used as a potential oral health care agent in the precaution of periodontal pathogens against *P. gingivalis* infection.

## Figures and Tables

**Table 1:** Inhibition zones of the light and dark-roast coffee extracts against *Porphyromonas gingivalis* (ATCC 33277) at 16 h.

Coffee Concentration (g/mL)	Light Roast				Dark Roast				Distilled Water ^†^
	0.03	0.07	0.17	0.33	0.03	0.07	0.17	0.33	
Diameter mean (SD) (mm)	0 (0)	8.4 (0.5)	10.6 (0.5)	12.8 (0.8)	0 (0)	7.9 (0.8)	10.3 (0.7)	12.5 (0.8)	0 (0)

^†^ Sterilized double-distilled water as the negative control group presented in the table.
